# Supplementary figures and images for: Health Literacy among Japanese College Students: Association with Healthy Lifestyle and Subjective Health Status
Source: Healthcare (Basel). 2023 Feb 27;11(5):704. doi: 10.3390/healthcare11050704 (PMC10000655; doi:10.3390/healthcare11050704)

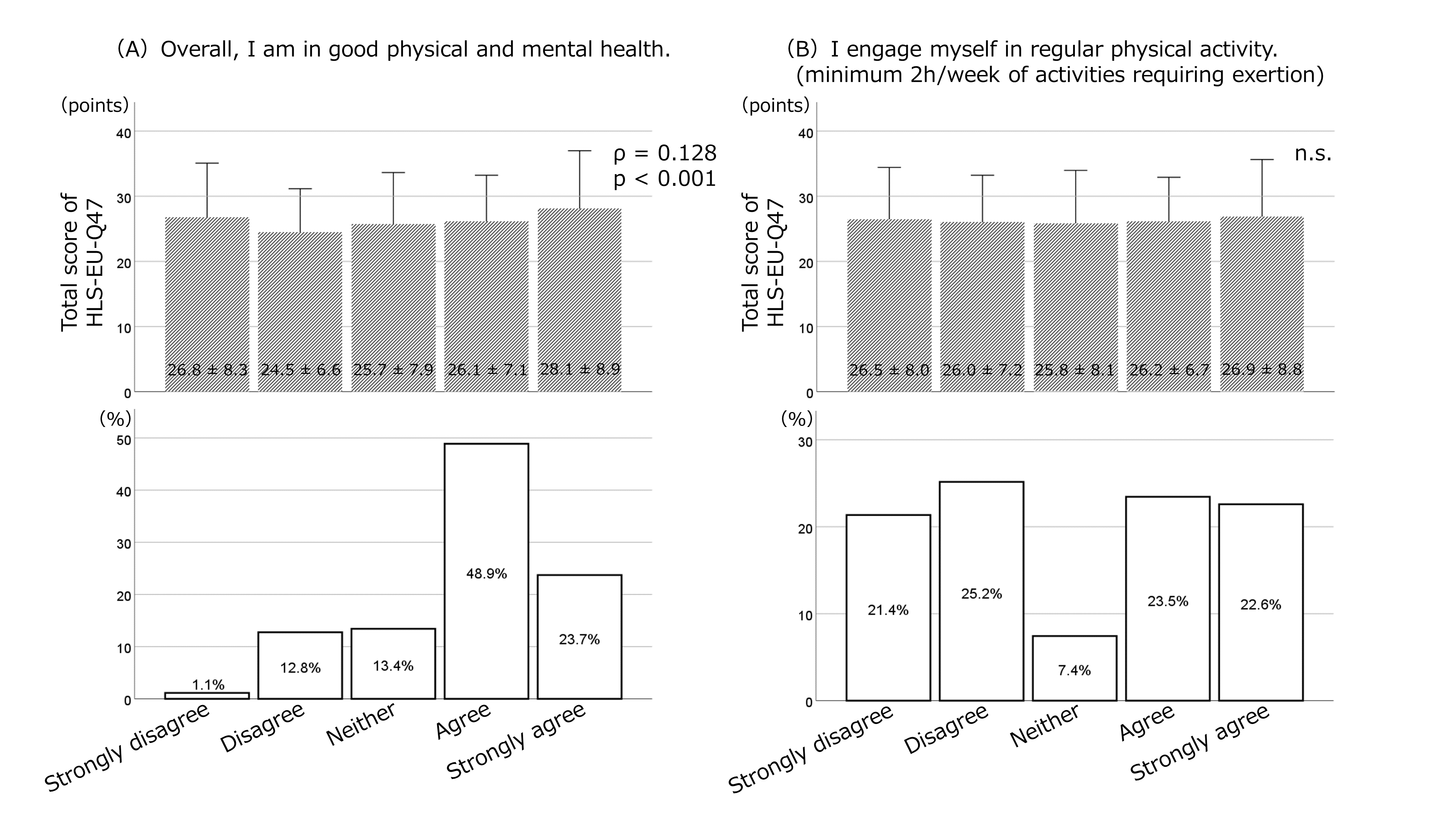

Supplement: Supplementary file 1 [file healthcare-11-00704-s001.zip › Figure S1AB.PNG]

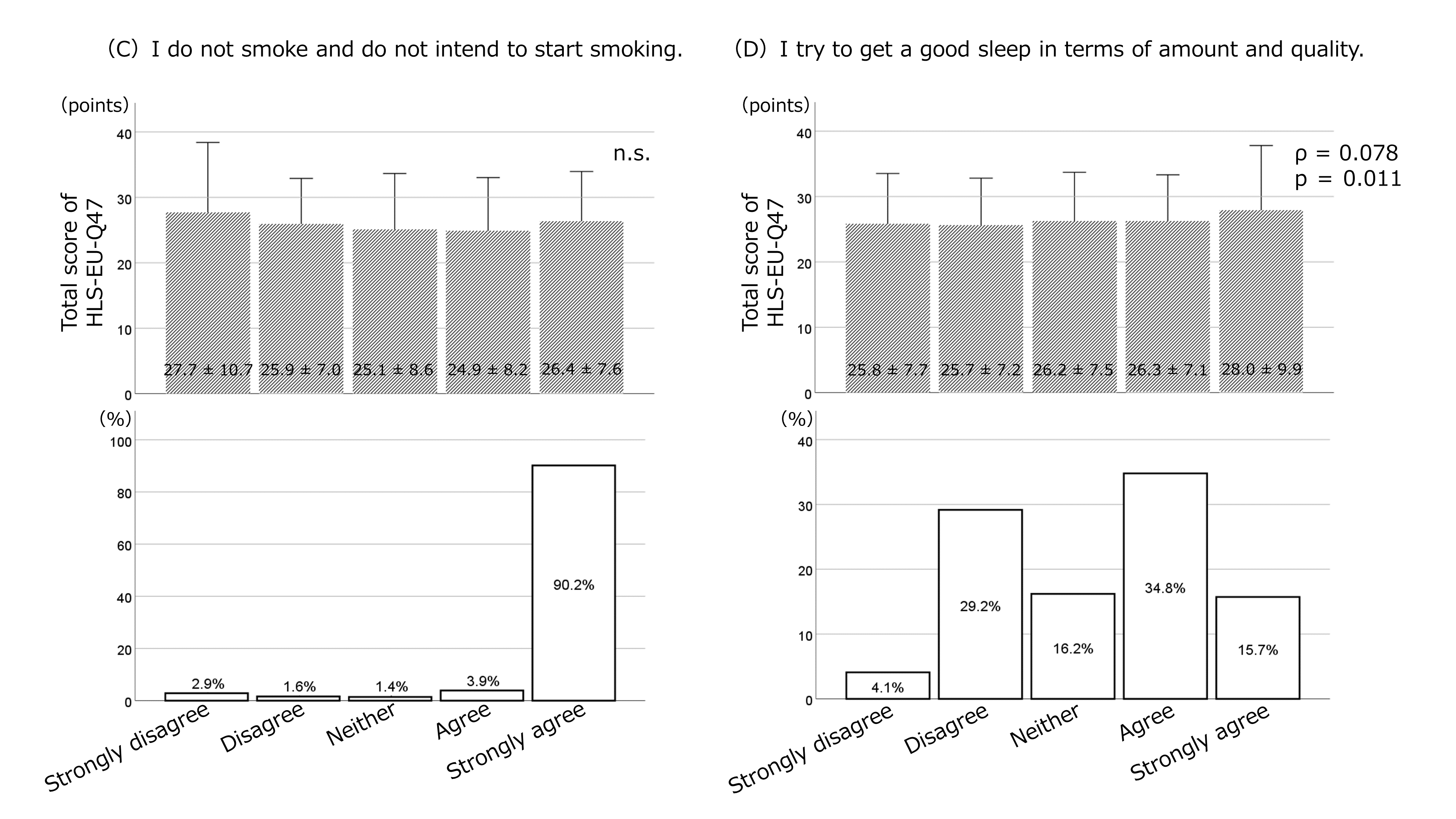

Supplement: Supplementary file 1 [file healthcare-11-00704-s001.zip › Figure S1CD.PNG]

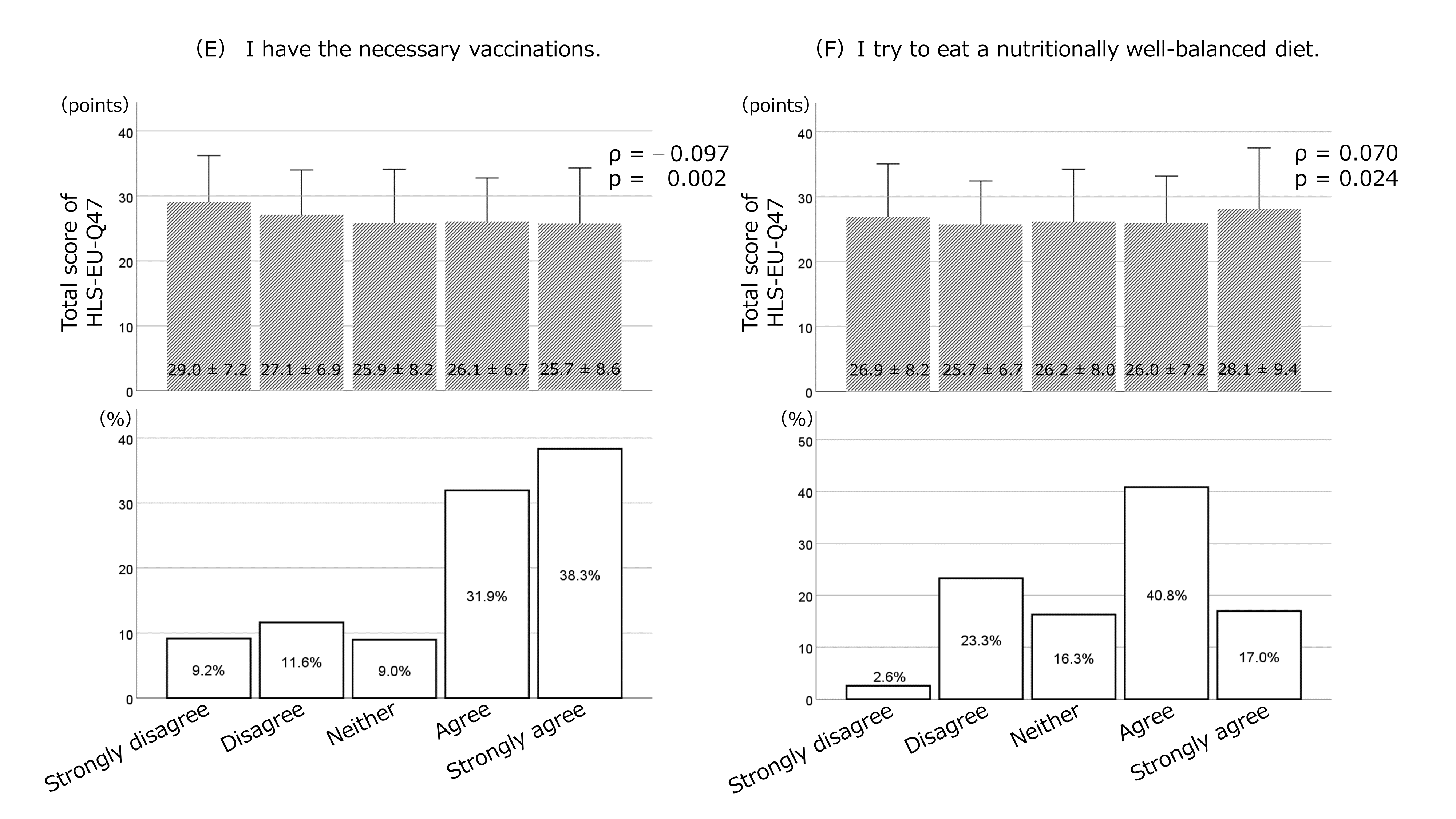

Supplement: Supplementary file 1 [file healthcare-11-00704-s001.zip › Figure S1EF.PNG]

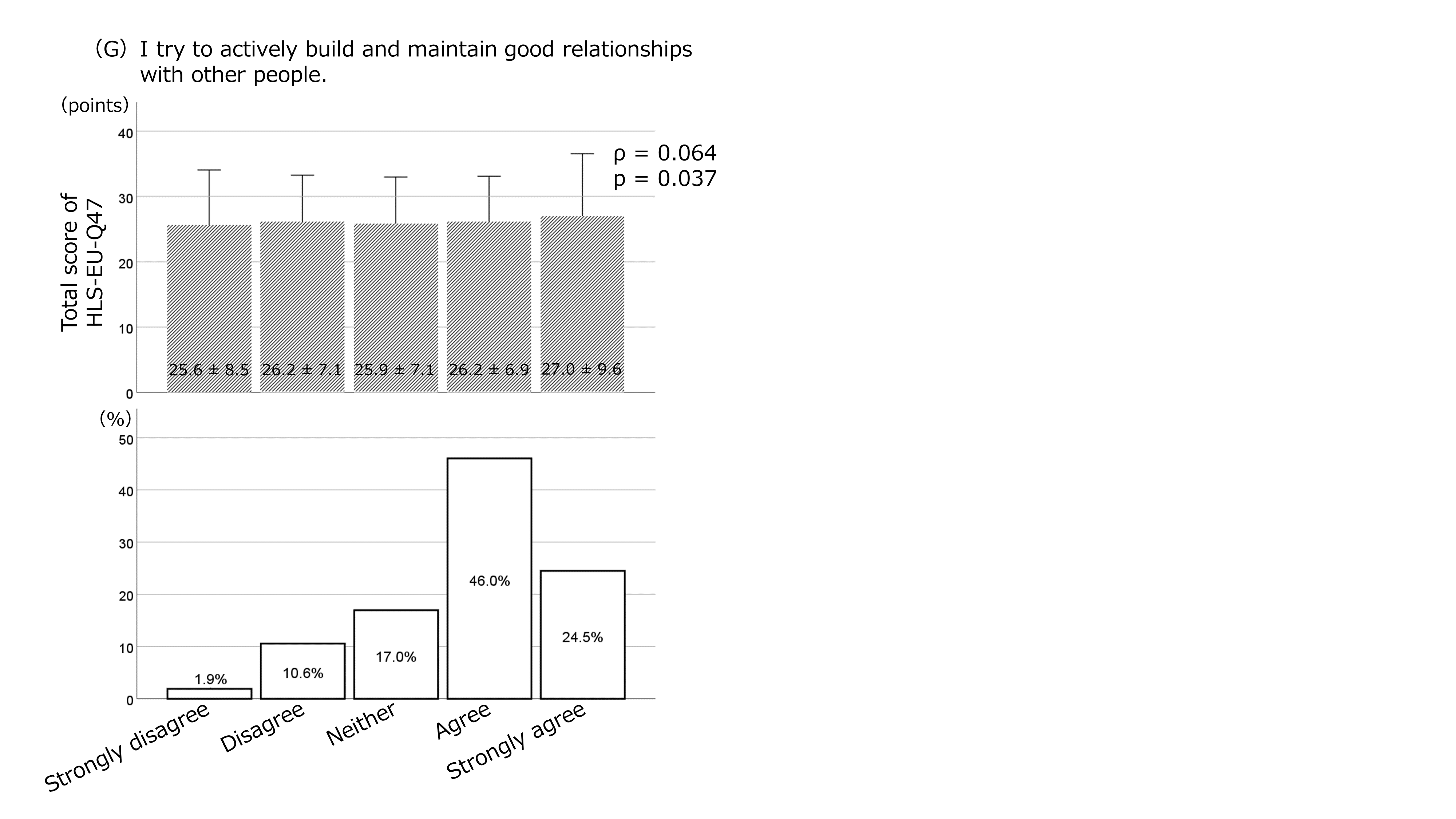

Supplement: Supplementary file 1 [file healthcare-11-00704-s001.zip › Figure S1G.PNG]

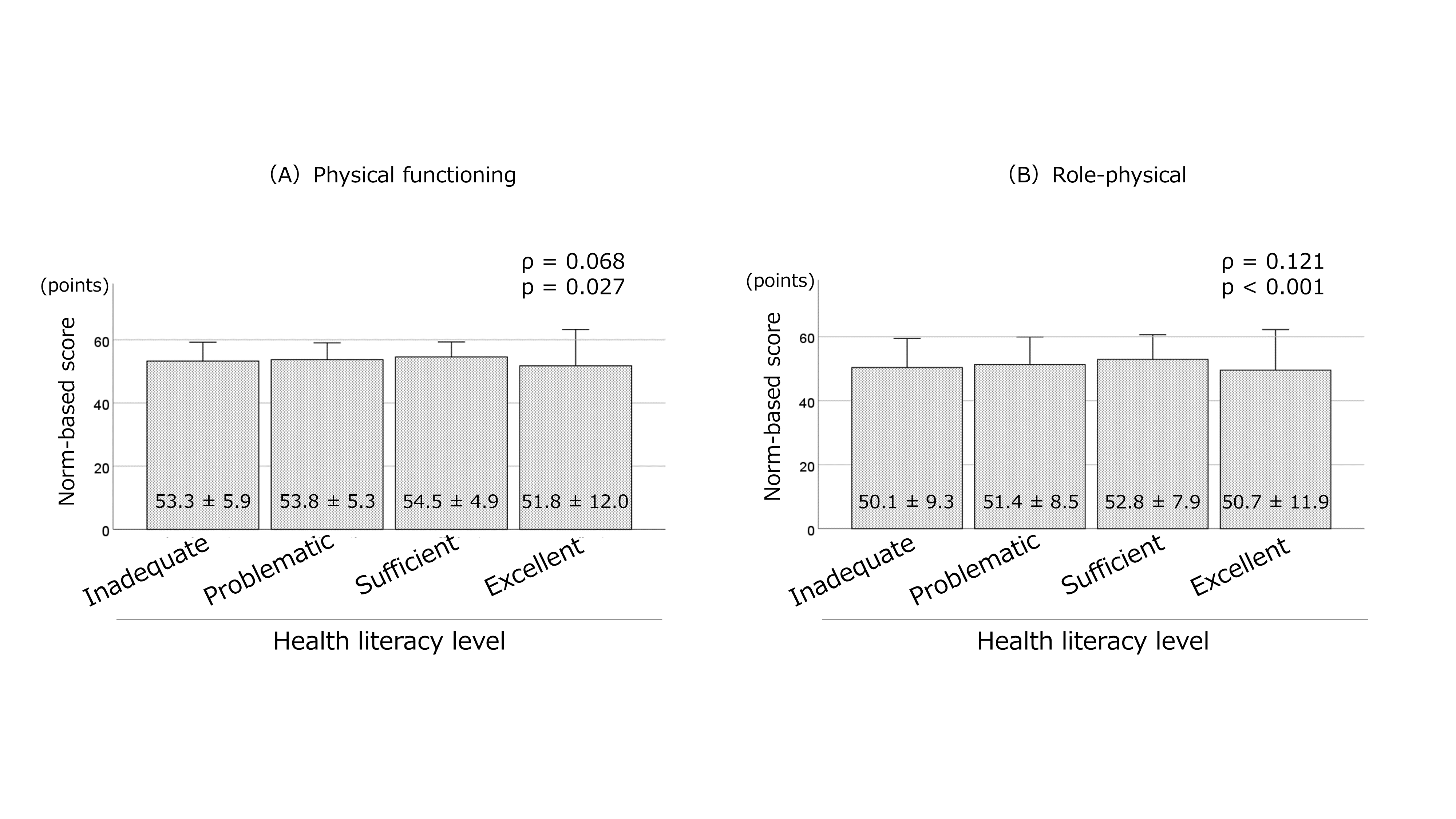

Supplement: Supplementary file 1 [file healthcare-11-00704-s001.zip › Figure S2AB.PNG]

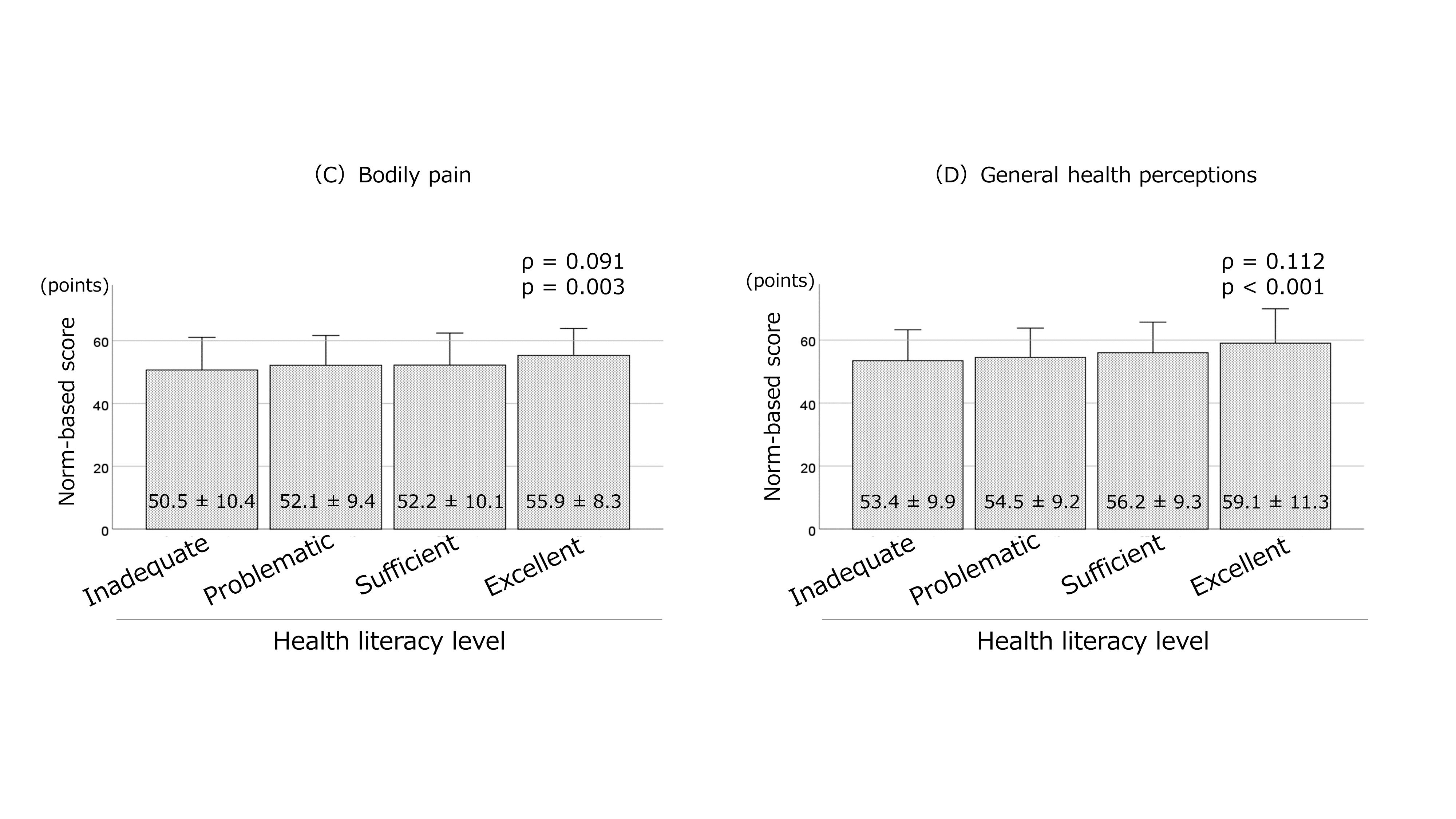

Supplement: Supplementary file 1 [file healthcare-11-00704-s001.zip › Figure S2CD.PNG]

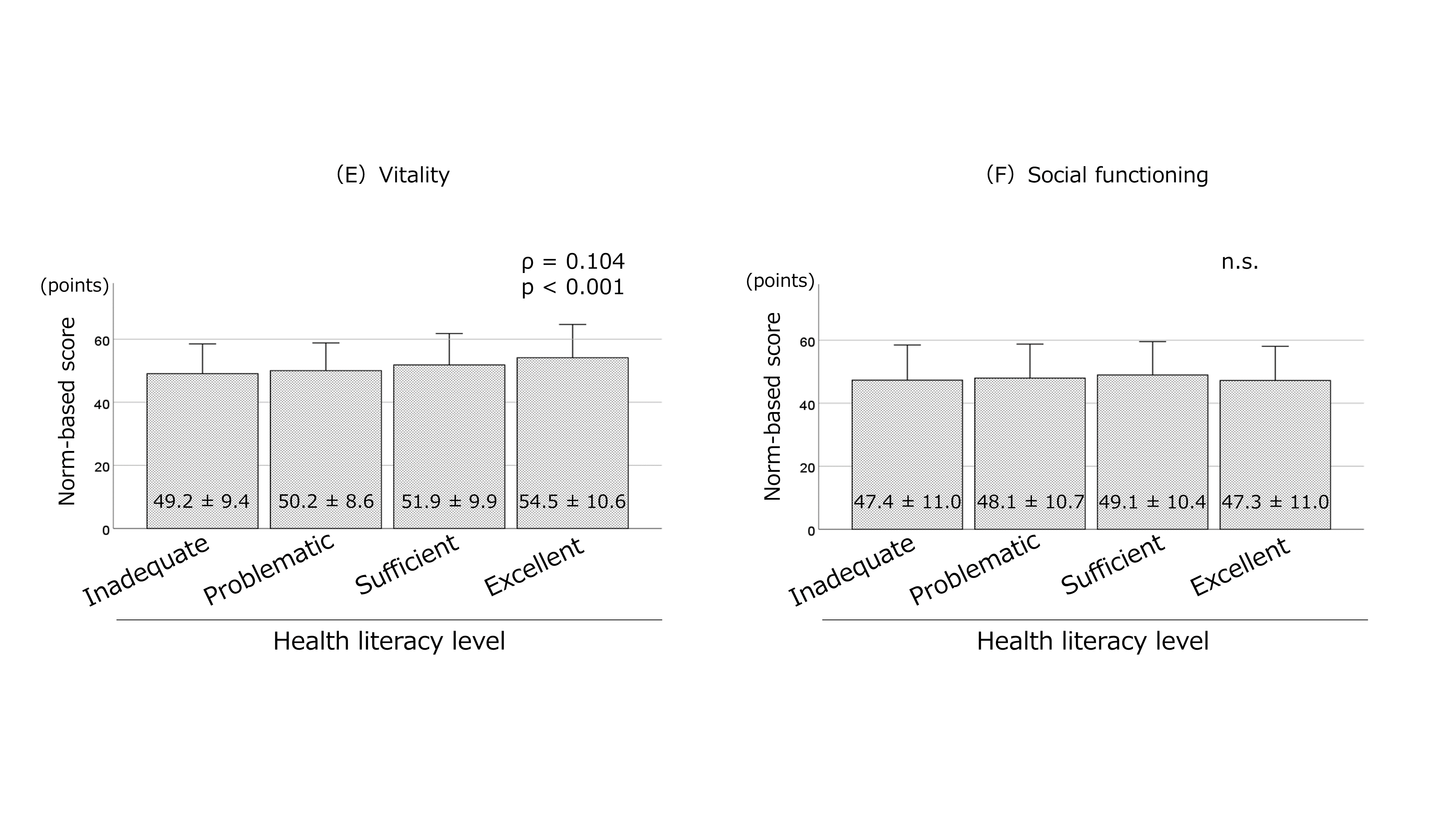

Supplement: Supplementary file 1 [file healthcare-11-00704-s001.zip › Figure S2EF.PNG]

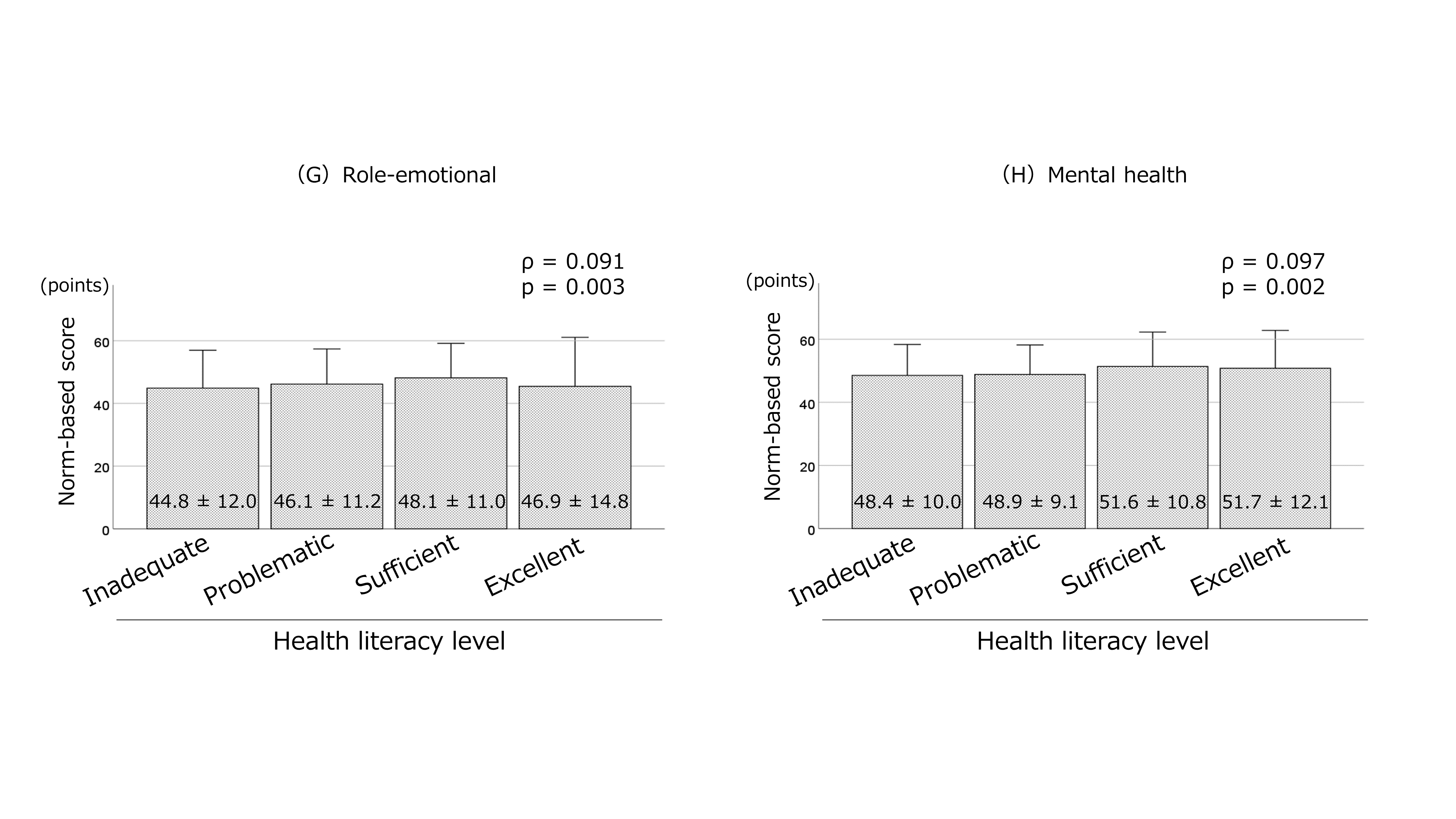

Supplement: Supplementary file 1 [file healthcare-11-00704-s001.zip › Figure S2GH.PNG]
